# Renal stress-associated extracellular vesicle signaling linked to lysosomal cathepsin D and NEK7–NLRP3 neuroinflammation

**DOI:** 10.1186/s43556-026-00608-z

**Published:** 2026-10-12

**Authors:** Gunhyuk Park, Ho-Ryong Yoo, Hye-Sun Lim

**Affiliations:** 1 Herbal Medicine Resources Research Center, Korea Institute of Oriental Medicine, 111 Geonjae-Ro, Naju-Si, Jeollanam-Do 58245 Republic of Korea; 2 Department of Cardiology and Neurology, College of Korean Medicine, Daejeon University, Daejeon, 34520 Republic of Korea

**Keywords:** MPTP, NLRP3 inflammasome, Neuroinflammation, Parkinson’s disease, Extracellular vesicles

## Abstract

Systemic exposure to neurotoxins such as 1-methyl-4-phenyl-1,2,3,6-tetrahydropyridine (MPTP) affects both the central nervous system (CNS) and peripheral organs; however, the contribution of peripheral organ stress to neuroinflammation remains incompletely understood. Here, we propose a peripheral-to-central signaling mechanism in which renal stress–associated extracellular vesicles (EVs) are linked to microglial inflammasome activation. Using computational and biochemical approaches, cathepsin D (CTSD) was identified as a molecular target of the MPTP metabolite 1-methyl-4-phenylpyridinium (MPP +). MPP + accumulated in renal tissue and was associated with lysosomal stress and tubular injury, accompanied by increased release of circulating EVs enriched with renal injury markers. Pharmacological inhibition of pepstatin A-sensitive lysosomal protease activity attenuated renal injury and EV release. MPTP exposure was also associated with blood–brain barrier disruption, potentially permitting circulating EV-associated signaling to influence the CNS. Plasma-derived EVs isolated under MPTP-treated conditions were associated with increased microglial activation, whereas pharmacological inhibition of EV biogenesis attenuated this response. Mechanistically, EV exposure was associated with inflammatory responses involving NIMA-related kinase 7 (NEK7)–NOD-like receptor family pyrin domain-containing 3-associated inflammatory signaling and IL-1β maturation. Consistent with these findings, NEK7 deficiency attenuated microglial activation and dopaminergic neuron loss in vivo. Together, these findings suggest that renal stress-associated EVs may be involved in linking peripheral stress to central neuroinflammatory responses. This study provides a tentative but verifiable framework for renal stress-EV-inflammasome communication in toxin-induced neuroinflammation.

**Supplementary Information:**

The online version contains supplementary material available at https://doi.org/10.1186/s43556-026-00608-z.

## Introduction

Parkinson’s disease (PD) is a progressive neurodegenerative disorder and a major cause of disability worldwide [1, 2]. Although the cardinal features of PD arise from the loss of dopaminergic neurons in the substantia nigra, its pathobiology likely extends beyond neuron-autonomous mechanisms to involve neuroinflammation and vascular dysfunction [1]. Microglial activation, increased production of proinflammatory cytokines, and disruption of the blood–brain barrier (BBB) have been consistently reported in patients and experimental models [1, 2]. Despite advances in symptomatic therapies, disease-modifying interventions remain limited, underscoring the need to identify upstream regulators of central immune activation.

Among innate immune pathways, the NLRP3 inflammasome serves as a central signaling hub that translates cellular stress into caspase-1 activation and interleukin-1β (IL-1β) maturation [3, 4]. NIMA-related kinase 7 (NEK7) has been identified as an essential licensing factor for NOD-like receptor family pyrin domain-containing 3 (NLRP3) inflammasome assembly, positioning the NEK7-NLRP3 axis as a critical regulator of microglial inflammatory activation [4–6]. In toxin-based PD models, including those induced by 1-methyl-4-phenyl-1,2,3,6-tetrahydropyridine (MPTP), the BBB integrity is frequently compromised, creating a permissive environment wherein circulating factors can access and influence the central nervous system (CNS) [7–9]. Clinical and epidemiological evidence suggests that PD is accompanied by systemic alterations, including changes in renal function and related biomarkers [10, 11]. Reduced kidney function is associated with an increased PD risk, and mild alterations in renal parameters have been reported in such patients [10, 12]. The pathological significance of these findings remains unclear; however, peripheral organ stress may contribute to disease processes beyond the CNS.

Extracellular vesicles (EVs) are increasingly recognized as key mediators of interorgan communication, transporting proteins, lipids, and nucleic acids that reflect the physiological or pathological state of their cells of origin [13, 14]. Depending on their molecular cargo, EVs can either amplify or resolve inflammatory responses, suggesting that their functional impact is highly context-dependent [14, 15]. The kidney is a central organ in systemic homeostasis and detoxification and is particularly susceptible to circulating toxins and metabolic stressors. Renal stress may influence distant organs through systemic signaling pathways, including EV-mediated mechanisms, and renal tubular epithelial cells are particularly enriched in lysosomal activity because of their high metabolic and endocytic burden, rendering them highly vulnerable to toxic and inflammatory stress [16–18]. However, the potential contribution of renal stress-associated EVs to neuroinflammatory processes remains unclear. Several studies have further suggested that EVs may cross or influence the BBB under pathological conditions, thereby contributing to peripheral-to-central inflammatory communication [19–22]. Among lysosomal proteases, cathepsin D (CTSD) has been implicated in lysosomal stress responses, inflammatory signaling, and neurodegenerative processes, although its role in peripheral organ-brain inflammatory communication remains unclear.

MPTP models are commonly used to study dopaminergic neurodegeneration; however, both MPTP and its active metabolite 1-methyl-4-phenylpyridinium (MPP⁺) undergo systemic distribution and peripheral metabolism [23, 24]. Additionally, BBB disruption observed in these models provides a context in which circulating factors may access the brain parenchyma [8, 25]. These features suggest that systemic MPTP exposure may involve direct neurotoxicity and peripheral contributions to central inflammatory responses. However, whether renal stress-associated EVs generated following systemic MPTP exposure contribute to neuroinflammation, particularly through microglial NEK7-NLRP3 signaling, remains unclear. Therefore, we hypothesized that systemic MPTP exposure induces renal stress-associated EV signaling that contributes to microglial NEK7-NLRP3 inflammasome activation and downstream neuroinflammatory responses.

Therefore, we investigated whether systemic MPTP exposure is associated with a peripheral organ-brain signaling framework involving circulating EVs that contributes to microglial inflammasome activation and IL-1β-associated neurotoxicity. Specifically, we characterized circulating EVs associated with renal injury following MPTP administration, evaluated their potential to modulate microglial inflammatory signaling with a focus on the NEK7-NLRP3 axis, and assessed whether pharmacological modulation of EV release or inflammasome-associated inflammatory signaling could attenuate downstream neuroinflammatory responses.

## Results

### Computational and experimental analyses identify CTSD as a renal-associated target of MPP +

**Fig. 1:**
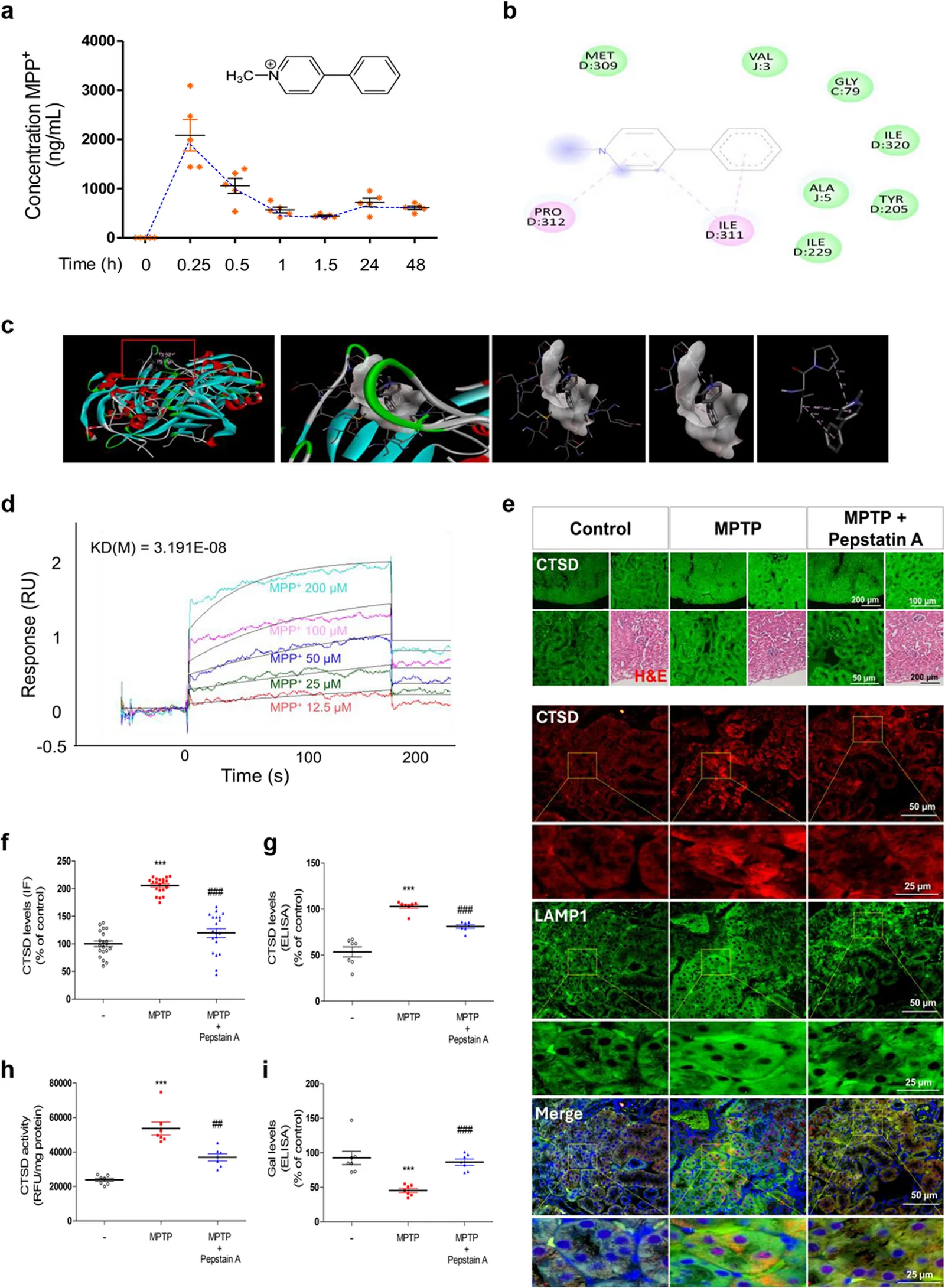
Systemic MPTP exposure is associated with renal accumulation of MPP^+^ and CTSD-associated lysosomal stress responses. **a** Renal concentration–time profile of MPP^+^ following systemic MPTP administration, demonstrating rapid renal accumulation of MPP^+^. **b**-**c** Representative docking poses of MPP^+^ within the predicted CTSD binding pocket. Two-dimensional interaction map of the top-ranked MPP^+^–CTSD docking configuration, showing major interacting residues and AutoDock Vina score. **d** SPR sensorgrams demonstrating concentration-dependent binding of MPP^+^ to immobilized CTSD and the calculated equilibrium dissociation constant (KD). MPTP-associated increase in renal CTSD enzymatic activity and lysosomal stress were partially attenuated by pepstatin A and CTSD silencing. **f**-**e** Representative renal images showing CTSD immunofluorescence staining, hematoxylin and eosin (H&E) staining, LAMP1 immunofluorescence staining, and merged images from control, MPTP, and MPTP + pepstatin A-treated mice. **g**-**h** Quantification of CTSD fluorescence intensity, protein expression, and enzymatic activity in kidney tissues. **i** Quantification of galectin-3 (Gal) levels. Data are presented as mean ± SEM. Statistical analysis was performed using one-way ANOVA followed by Tukey’s post-hoc test. ^***^P < 0.001 vs. Control; ^##^*P* < 0.01 and ^###^*P* < 0.001 vs. MPTP

To identify potential molecular mediators associated with MPP^+^-induced renal stress, transcriptomic comparison using the Connectivity Map (CMap) perturbagen database and STRING network analysis identified CTSD as a highly probable target with strong renal enrichment (Table S1 and Fig. S1). Consistent with these predictions, LC–MS/MS analysis demonstrated rapid renal accumulation of MPP^+^ following systemic MPTP administration (Fig. 1a). MPP^+^ was undetectable in control tissues but reached concentrations of approximately 400–3000 ng/mL in MPTP-treated kidneys, supporting substantial renal deposition and retention following systemic exposure. To further evaluate whether CTSD may directly interact with MPP^+^, molecular docking and SPR analyses were subsequently performed. Docking analysis demonstrated stable binding of MPP^+^ within the catalytic pocket of CTSD, supporting a structurally favorable interaction. SPR analysis further demonstrated direct concentration-dependent binding between MPP^+^ and CTSD with nanomolar-range affinity (KD = 3.19 × 10⁻⁸ M) (Fig. 1b-d), supporting the possibility that CTSD may represent a pharmacologically relevant renal-associated target of MPP^+^.

**Fig. 2:**
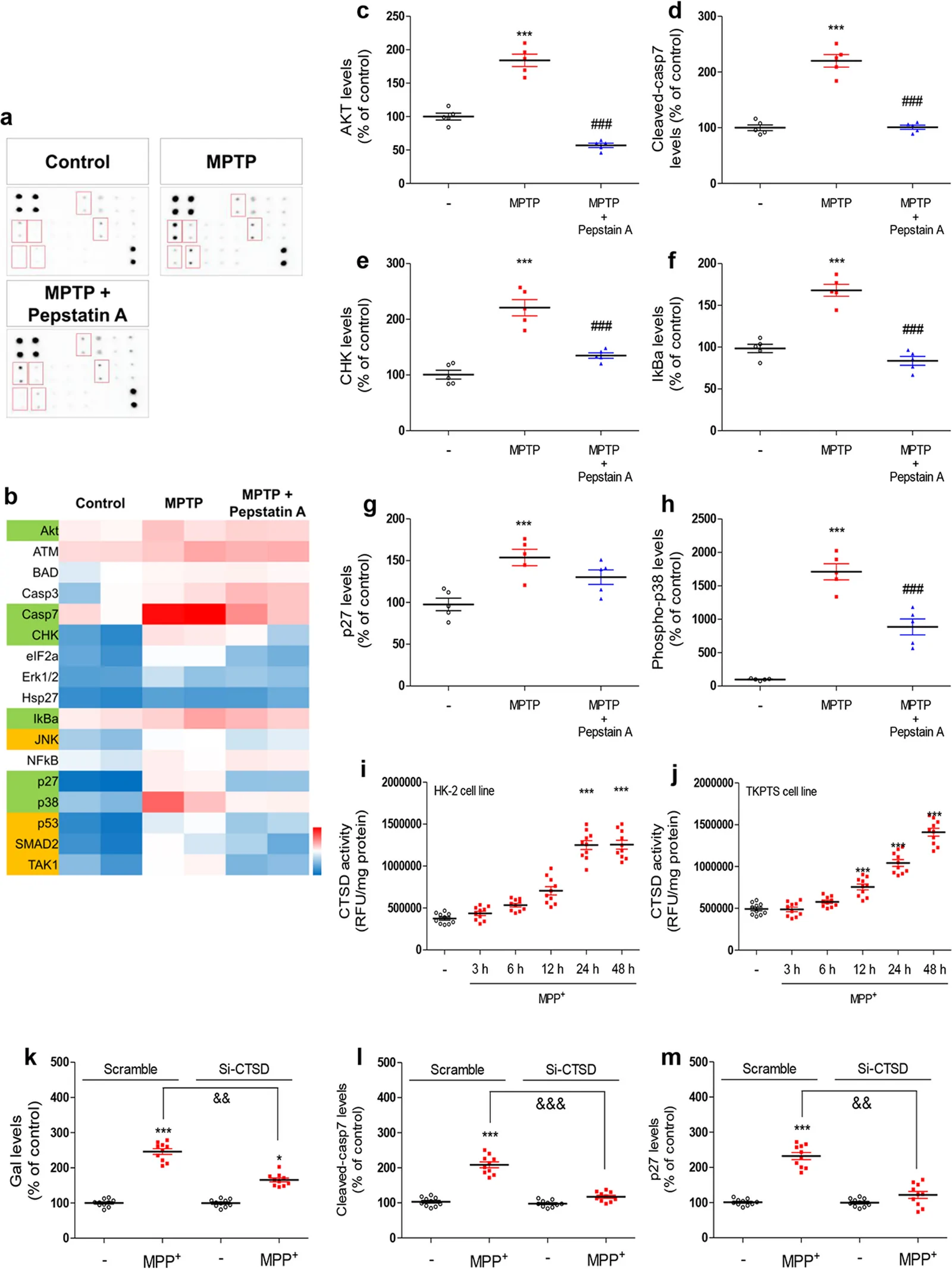
Representative stress-associated protein array membranes and corresponding heatmap analysis of differentially regulated signaling proteins in kidney lysates. **a**–**h** Quantitative analyses of selected signaling proteins, including AKT, cleaved-caspase-7, CHK, IκBα, p27, and phospho-p38. **i**-**j** Time-dependent changes in CTSD enzymatic activity following MPP^+^ treatment in HK-2 and TKPTS renal tubular cell lines. **k**–**m** Effects of siRNA-mediated CTSD knockdown on Gal-3, cleaved-caspase-7, and p27 levels following MPP^+^ exposure. Data are presented as mean ± SEM. Statistical analysis was performed using one-way ANOVA followed by Tukey’s post-hoc test. ^*^P < 0.05 and ^***^P < 0.001 vs. Control; ^###^*P* < 0.001 vs. MPTP; ^&&^*P* < 0.01 and ^&&&^*P* < 0.001 vs. MPTP scramble-transfected MPP^+^ group

To determine whether systemic MPTP exposure was associated with increased CTSD enzymatic activity and lysosome-associated alterations in vivo, histological and biochemical analyses were performed in MPTP-treated mice with or without pepstatin A treatment. Immunofluorescence and H&E staining demonstrated increased CTSD expression, lysosome-associated alterations, and tubular injury following MPTP exposure, whereas pepstatin A treatment partially attenuated these pathological changes (Fig. 1e-h). Additional line-scan overlap analyses are provided in Fig. S2. Consistently, Gal-3 expression was significantly elevated in MPTP-treated kidneys and reduced following pepstatin A administration (Fig. 1i). Protein array analysis further demonstrated broad activation of stress-associated signaling pathways under MPTP conditions, whereas pepstatin A treatment globally attenuated these alterations (Fig. 2a-h). CTSD enzymatic activity in HK-2 and TKPTS renal tubular cells further demonstrated time-dependent increases following MPP^+^ exposure (Fig. 2i-j). To genetically validate the involvement of CTSD, CTSD expression was silenced using siRNA in renal tubular cells (Fig. 2k-m). CTSD knockdown significantly attenuated MPP^+^-induced Gal-3 upregulation as well as stress-associated protein alterations, including cleaved-caspase-7 and p27 expression, supporting the involvement of CTSD-associated lysosomal stress signaling following MPP^+^ exposure. Together, these findings suggest that systemic MPTP exposure was associated with renal accumulation of MPP^+^, CTSD-associated lysosomal alterations, and activation of inflammatory and cellular stress responses, whereas pharmacological or genetic suppression of CTSD-associated signaling partially attenuated these pathological changes.

### MPTP-associated BBB disruption and CTSD-related inflammatory signaling were linked to enhanced central neuroinflammatory responses

**Fig. 3:**
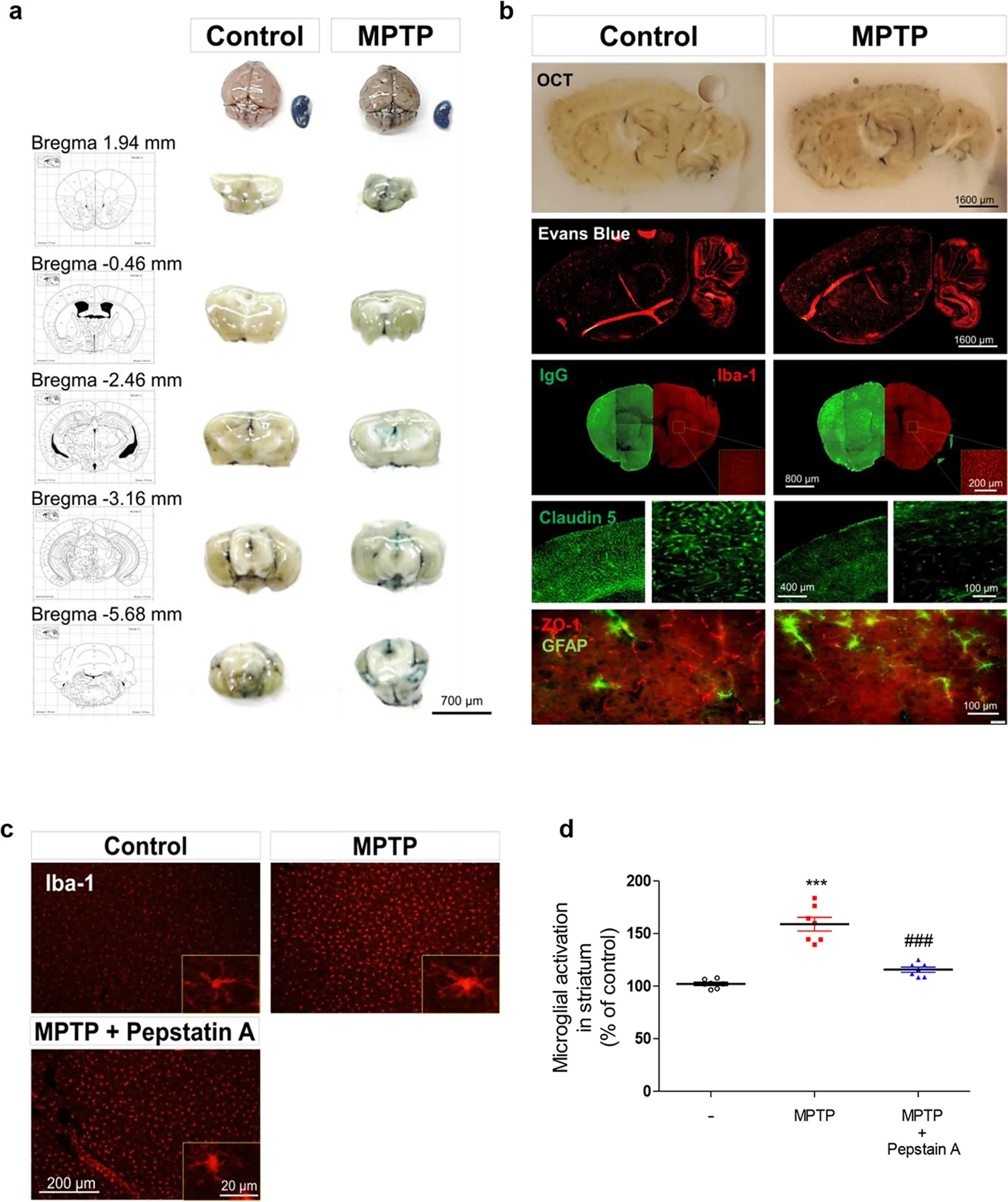
MPTP-induced BBB disruption and CTSD-associated inflammatory signaling are linked to enhanced neuroinflammatory responses. **a**-**b** Representative images showing BBB-associated alterations following MPTP exposure, including increased Evans blue extravasation, endogenous IgG leakage, reduced and discontinuous claudin-5 expression, altered ZO-1 distribution, and GFAP-positive astrocytic changes. **c**-**d** Representative Iba-1 immunofluorescence images and quantitative analysis of microglial activation in the striatum of control, MPTP, and MPTP + pepstatin A-treated mice. MPTP exposure increased microglial activation, whereas pepstatin A treatment partially attenuated these responses. Data are presented as mean ± SEM. Statistical analysis was performed using one-way ANOVA followed by Tukey’s post-hoc test. ^***^P < 0.001 vs. Control; ^###^*P* < 0.001 vs. MPTP

**Fig. 4:**
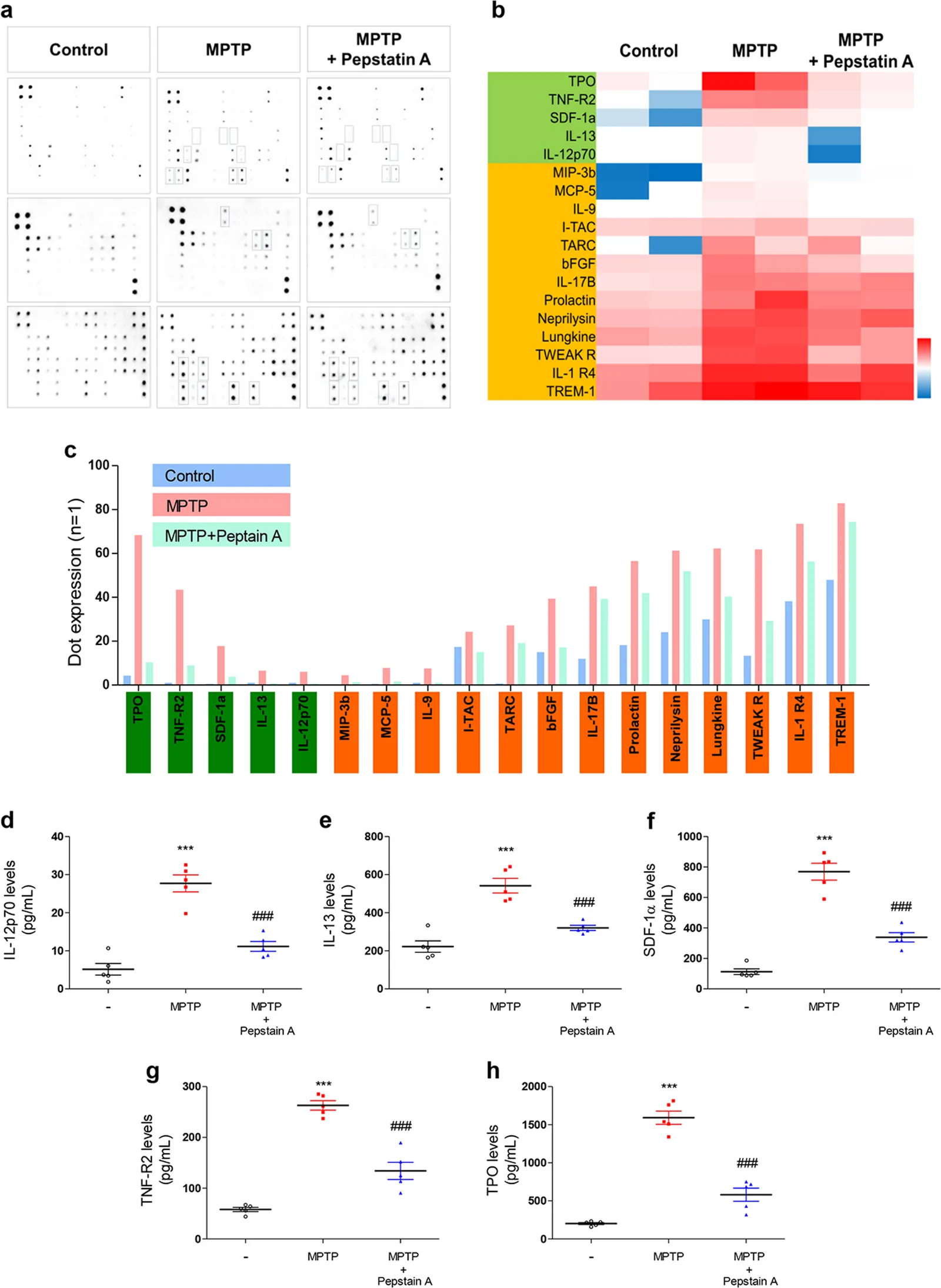
CTSD inhibition by pepstatin A attenuated MPTP-induced inflammatory cytokine and chemokine responses in the substantia nigra pars compacta (SNpc). **a** Representative cytokine/chemokine antibody array membranes obtained from SNpc lysates of Control, MPTP, and MPTP + pepstatin A-treated mice. Differentially regulated inflammatory mediators are highlighted by blue boxes. **b** Heatmap visualization of relative cytokine and chemokine expression profiles across experimental groups. Red indicates relative upregulation, whereas blue indicates relative downregulation. **c** Semi-quantitative densitometric analysis of selected cytokine/chemokine array signals. **d**–**h** ELISA-based validation of representative inflammatory mediators, including IL-12p70, IL-13, SDF-1α, TNF-R2, and TPO, in SNpc lysates. MPTP treatment significantly increased the expression of multiple inflammatory mediators, whereas pepstatin A administration markedly attenuated these responses. Data are presented as mean ± SEM. Statistical significance was determined using one-way ANOVA followed by Tukey’s post-hoc test. ^***^P < 0.001 vs. Control; ^###^*P* < 0.001 vs. MPTP

To investigate whether systemic MPTP exposure was associated with BBB disruption and subsequent neuroinflammatory alterations, vascular permeability, tight-junction integrity, microglial activation, and inflammatory cytokine responses were evaluated in the brains of treated mice. Previous studies have reported that MPTP disrupts the BBB, and our findings further support this phenomenon. Following MPTP administration (30 mg/kg/day, i.p.), increased Evans Blue extravasation and altered tight-junction-associated marker distribution were observed in the brains of MPTP-treated mice, consistent with enhanced BBB permeability (Fig. 3a). Increased IgG leakage and GFAP-associated structural alterations further supported BBB-associated inflammatory changes under MPTP conditions (Fig. 3b). To further determine whether CTSD-associated renal stress responses were linked to central inflammatory alterations following BBB disruption, microglial activation and inflammatory cytokine profiles were subsequently examined. In the control group, Iba-1-positive microglia were sparse and exhibited a ramified morphology with elongated processes, indicative of a resting state (Fig. 3c). In contrast, MPTP (30 mg/kg/day, i.p.)-treated mice showed a marked increase in Iba-1-positive cell density, accompanied by enlarged soma and retracted processes, hallmarks of activated microglia. Treatment with pepstatin A (20 mg/kg/day, i.p.), an inhibitor of aspartic lysosomal proteases including CTSD, partially attenuated these morphological inflammatory alterations and reduced microglial activation (Fig. 3c-d). Collectively, these findings suggest that MPTP-induced microglial activation was associated with increased CTSD-related inflammatory responses and that pharmacological inhibition with pepstatin A partially attenuated neuroinflammatory alterations in the CNS. Cytokine protein array analysis using SNpc samples demonstrated broad increases in proinflammatory cytokines and chemokines following MPTP exposure (Fig. 4a and Fig. S3). Representative cytokine array membranes and heatmap analyses supported these inflammatory expression patterns (Fig. 4b-c). Selected cytokines showing prominent alterations in the array analysis, including IL-12p70, IL-13, SDF-1α, TNF-R2, and TPO, were further validated by ELISA (Fig. 4d-h). In contrast, co-treatment with pepstatin A broadly attenuated these inflammatory alterations, consistent with reduced neuroinflammatory signaling in the SNpc. Together, these findings suggest that systemic MPTP exposure was associated with BBB disruption, enhanced peripheral access to the brain parenchyma, microglial activation, and increased inflammatory cytokine responses, whereas pharmacological inhibition of CTSD-associated lysosomal signaling partially attenuated these neuroinflammatory alterations.

### MPTP-induced renal stress was associated with altered circulating EV profiles and neuroinflammatory responses

**Fig. 5:**
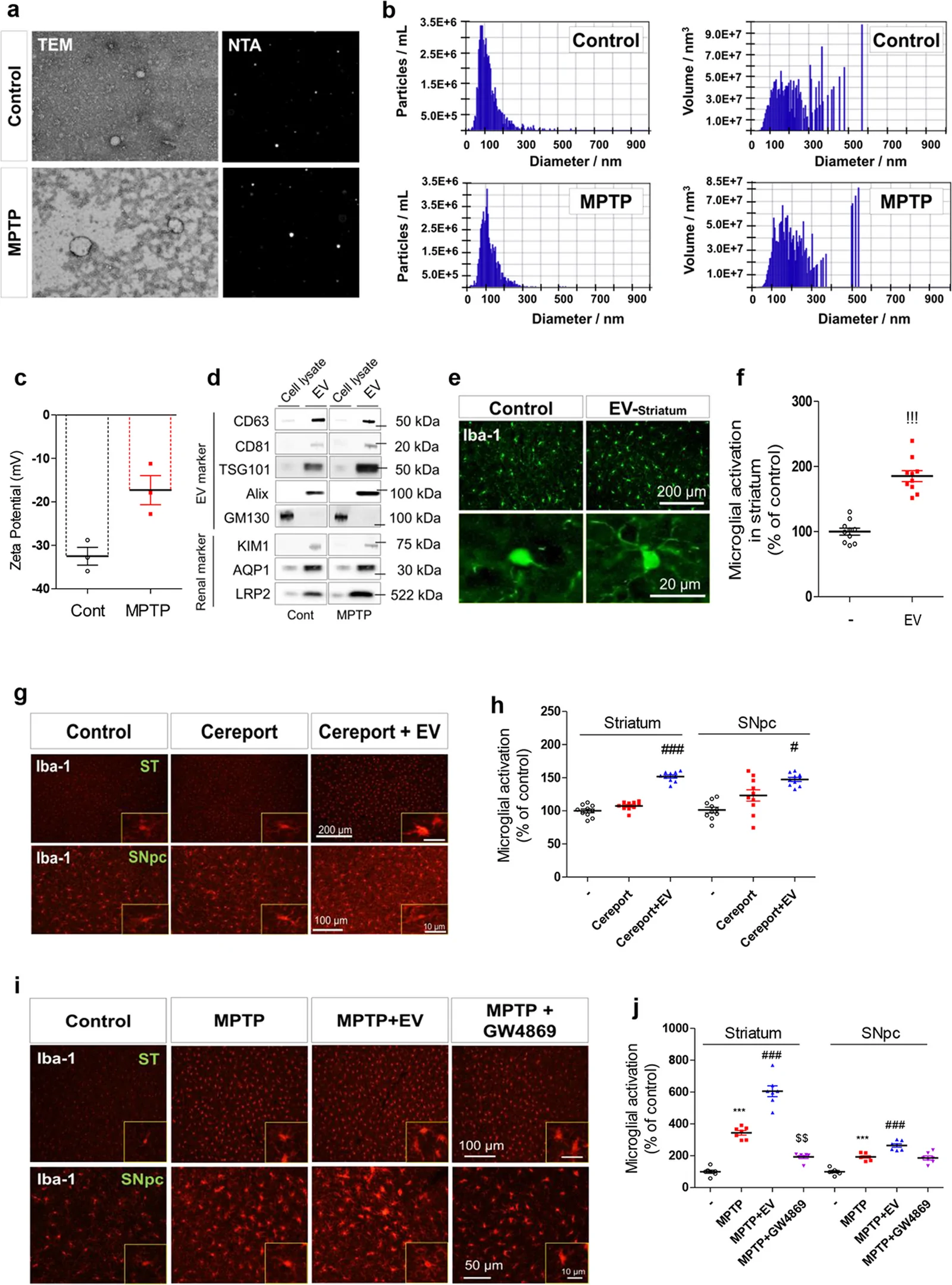
Renal stress-associated circulating extracellular vesicles are associated with MPTP-induced microglial activation in the brain. **a**–**b** Characterization of plasma-derived extracellular vesicles (EVs) isolated from control and MPTP-treated mice. Representative TEM images and nanoparticle tracking analysis (NTA) profiles showing EV particle size and distribution are presented. **c** Zeta potential measurements are shown. **d** Western blot characterization of EV-associated markers (CD63, CD81, TSG101, and Alix) and kidney injury-associated proteins (KIM-1, AQP1, and LRP2) in isolated EV fractions. GM130 was used as a negative control marker. **e**–**f** Representative Iba-1 immunostaining and quantification of microglial activation in the striatum following intra-striatal stereotactic injection of plasma-derived EVs. **g**–**h** Representative Iba-1 staining and quantification of microglial activation in the striatum (ST) and substantia nigra pars compacta (SNpc) following cereport-mediated BBB opening and EV administration. **i**–**j** Representative Iba-1 staining and quantification showing the effects of GW4869-mediated EV release inhibition on MPTP-induced microglial activation in the ST and SNpc. Data are presented as mean ± SEM. Statistical analysis was performed using one-way ANOVA followed by Tukey’s post-hoc test. ^***^P < 0.001 and ^!!!^*P* < 0.001 vs. Control; ^#^*P* < 0.05, ^###^*P* < 0.001 vs. MPTP; ^$$^*P* < 0.01 vs. MPTP + EV

To determine whether systemic MPTP exposure was associated with alterations in circulating EV characteristics, plasma-derived EV fractions were analyzed using ultrastructural, biophysical, and biochemical approaches. TEM and nanoparticle tracking analyses demonstrated increased EV abundance and altered particle distributions following MPTP exposure, with broader size heterogeneity observed under MPTP conditions (Fig. 5a-b and Supplementary Video). Zeta potential analysis further demonstrated altered surface electrostatic properties of circulating EVs following MPTP exposure (Fig. 5c).

Immunoblot analysis was performed to further characterize the circulating EV fractions (Fig. 5d and Fig. S4). Canonical EV markers including CD63, CD81, TSG101, and Alix were detected in EV fractions, whereas the Golgi-resident protein GM130 was absent, supporting enrichment of EV-associated components with minimal cellular contamination. In addition, renal injury-associated proteins including KIM-1, AQP1, and LRP2 were more prominently detected in EV fractions isolated from MPTP-treated mice, suggesting an association between systemic MPTP exposure and renal stress-related EV alterations.

To further investigate whether these circulating EVs were associated with neuroinflammatory responses under conditions of BBB permeability, both intracerebral and intravenous EV administration experiments were performed. Stereotactical injection of MPTP-derived EVs (plasma EVs: 10 μg/3 μL) into the striatum increased Iba-1 immunoreactivity and was associated with morphological alterations in microglia, including enlarged soma and shortened processes (Fig. 5e-f). When the BBB was transiently opened by cereport administration (total volume: 0.75 mL), injection of MPTP-derived EVs (plasma EVs: 100 µg, i.v.) led to significant increases in Iba-1 immunoreactivity in both the SNpc and striatum, accompanied by hypertrophic microglial morphology (Fig. 5g-h). Similarly, intravenous administration of EVs following MPTP-induced BBB disruption resulted in elevated Iba-1 expression, indicating enhanced microglial activation. Treatment with GW4869 (3 mg/kg/day, i.p.), an inhibitor of EV biogenesis and release, markedly reduced Iba-1 levels compared with MPTP alone (Fig. 5i-j). Together, these findings suggest that systemic MPTP exposure was associated with altered circulating EV profiles enriched with renal injury-related markers and that these EVs were associated with enhanced microglial inflammatory activation under conditions of BBB permeability. In addition, pharmacological inhibition of EV biogenesis partially attenuated these neuroinflammatory responses, supporting a functional contribution of EV-associated signaling to peripheral-to-central inflammatory communication.

### NEK7-NLRP3-associated signaling mediated EV-related inflammatory responses in microglial cells

**Fig. 6:**
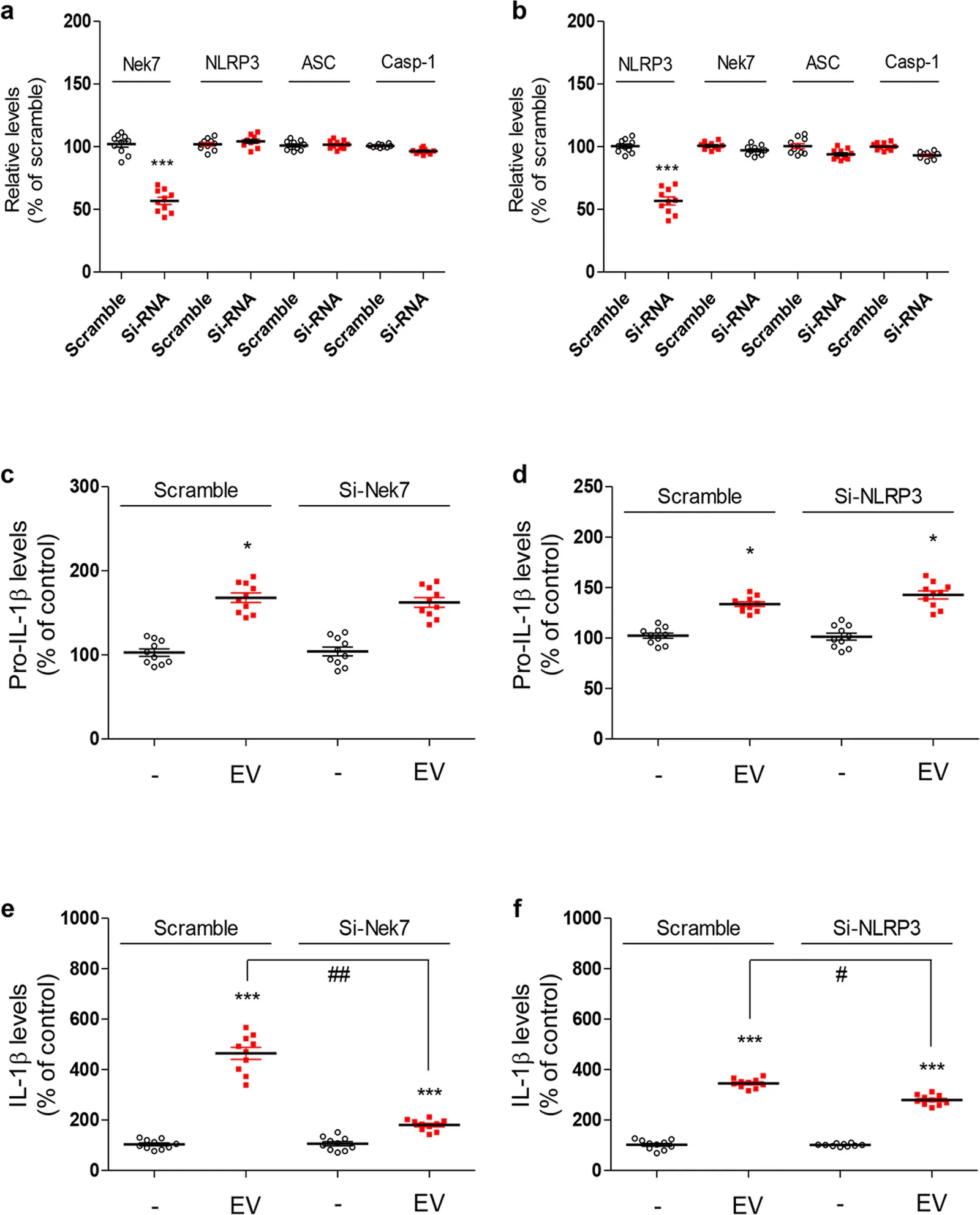
Kidney-associated EVs promote NEK7-NLRP3 inflammasome-associated IL-1β maturation in microglia. **a**-**b** siRNA specificity controls. Knockdown of NEK7 or NLRP3 in microglia reduces the intended target while leaving ASC and caspase-1 largely unchanged (relative to scramble; qPCR or immunoblot as indicated). **c**-**d** Pro-IL-1β (priming readout) after EV exposure. EVs increase pro-IL-1β, and this effect is minimally affected by si-NEK7 or si-NLRP3, indicating that priming is upstream/independent of NEK7–NLRP3. **e**–**f** Mature IL-1β secretion (activation readout). EV-induced IL-1β maturation is markedly reduced by si-NEK7 or si-NLRP3, supporting the involvement of NEK7-dependent inflammasome activation. Data are presented as mean ± SEM. Statistical analysis was performed using one-way ANOVA followed by Tukey’s post-hoc test. ^*^P < 0.05, ^**^P < 0.01, ^***^P < 0.001 vs corresponding control; ^#^*P* < 0.05, ^##^*P* < 0.01 vs EV + scramble

To investigate whether EV-associated inflammatory responses were linked to NEK7–NLRP3 inflammasome signaling, BV2 microglial cells were transfected with siRNAs targeting either NEK7 or NLRP3 prior to EV exposure. qPCR analysis confirmed that NEK7 expression was reduced by more than 80% in the NEK7 siRNA group, whereas NLRP3 mRNA levels were significantly decreased in the NLRP3 siRNA group. ASC and caspase-1 expression levels remained relatively unchanged across groups (Fig. 6a-b). Following treatment with plasma-derived EVs isolated from MPTP-treated mice, scramble siRNA-transfected cells exhibited increased IL-1β protein levels, whereas pro-IL-1β expression showed relatively minor changes. In contrast, both NEK7 and NLRP3 silencing attenuated EV-associated IL-1β production, with NEK7-silenced cells showing a comparatively greater reduction under the present experimental conditions (Fig. 6c-f). Together, these findings support the involvement of NEK7-NLRP3-associated signaling in EV-mediated microglial inflammatory responses and suggest that EV-associated inflammasome activation may preferentially regulate IL-1β maturation rather than basal pro-IL-1β expression under the present experimental conditions.

### Partial loss of NEK7 attenuated EV-associated neuroinflammatory, dopaminergic, and behavioral alterations following systemic MPTP exposure

**Fig. 7:**
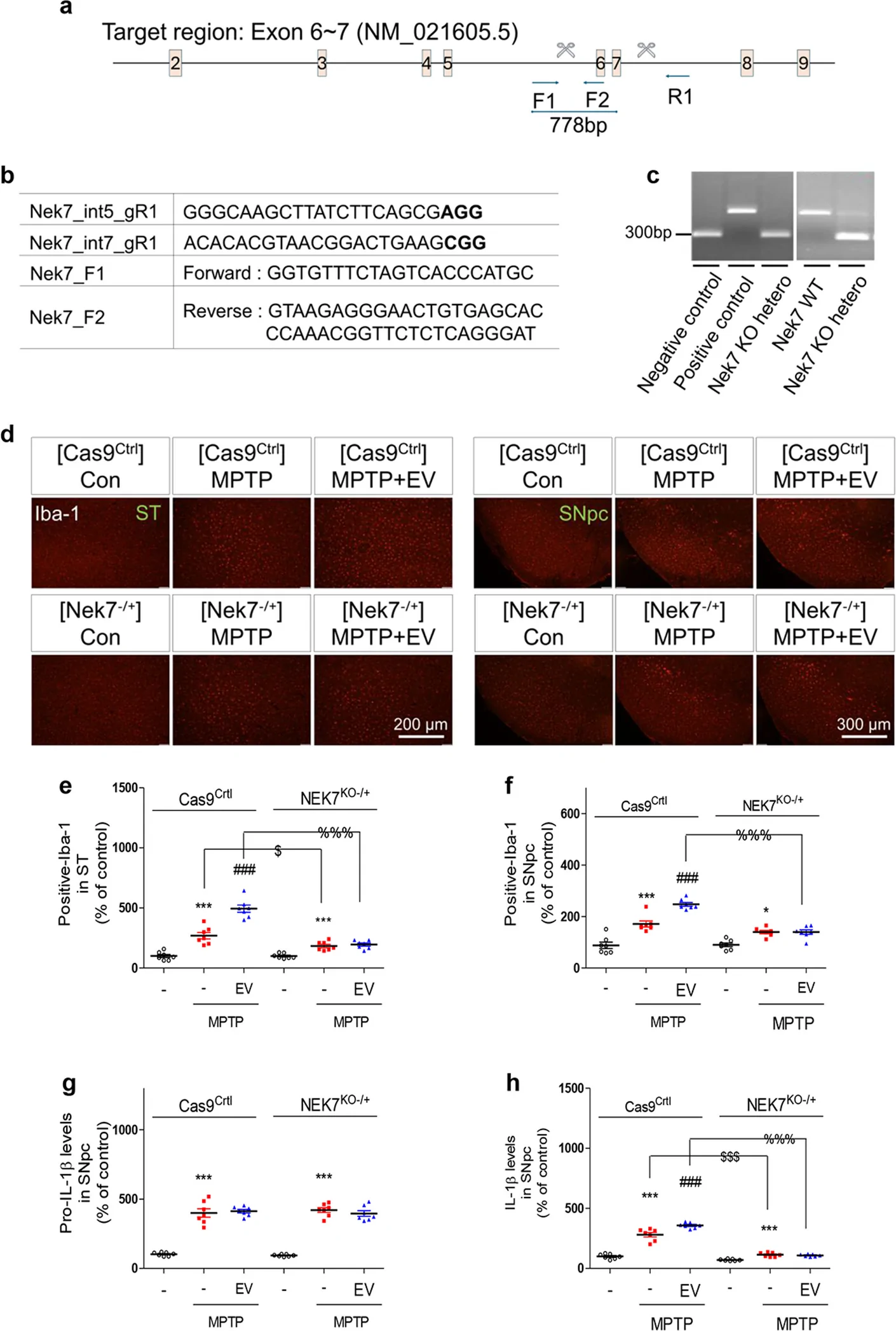
NEK7 haploinsufficiency attenuates EV-associated neuroinflammatory, dopaminergic, and behavioral alterations following systemic MPTP exposure. **a** CRISPR/Cas9 targeting strategy for NEK7, showing guide RNA target sites within exons 6–7 and positions of genotyping primers (F1, F2, and R1). **b** Sequences of guide RNAs and PCR primers used for NEK7 genome editing and genotyping. **c** Representative genotyping gel confirming NEK7^KO±^ mice. **d** Representative Iba-1 immunofluorescence images in the striatum (ST) and substantia nigra pars compacta (SNpc) from Cas9^Ctrl^ and NEK7^KO±^ mice across treatment groups (Control, MPTP, and MPTP + EV). **e**–**h** Quantitative analyses of Iba-1-positive microglial activation and inflammatory cytokine responses. NEK7 haploinsufficiency attenuated EV-enhanced microglial activation and IL-1β-associated inflammatory responses following MPTP exposure. Data are presented as mean ± SEM. Statistical analysis was performed using one-way ANOVA followed by Tukey’s post-hoc test. ^*^P < 0.05, ^***^P < 0.001 vs. corresponding Control group; ^###^*P* < 0.001 vs. MPTP-treated group; ^%%%^*P* < 0.001 vs. Cas9^Ctrl^ MPTP + EV group; ^$^*P* < 0.05 and ^$$$^*P* < 0.001 between the indicated groups

Genotyping PCR confirmed the successful generation of NEK7-deficient alleles (Fig. 7a-c). WT mice exhibited a single 778 bp band, whereas NEK7^KO^ mice displayed a 300 bp truncated fragment corresponding to the CRISPR-induced deletion. Heterozygous NEK7^KO+/–^ mice showed both bands, indicating coexistence of WT and deleted alleles. Because homozygous NEK7^KO−/–^ mice showed reduced viability, all subsequent experiments were performed using NEK7^KO+/–^ mice.

To evaluate the contribution of NEK7 signaling to EV-associated neuroinflammatory and neurodegenerative responses in vivo, NEK7^KO+/–^ mice and Cas9 control (Cas9^Ctrl^) mice were compared under MPTP (30 mg/kg, i.p.) and EV (100 µg, i.v.) exposure conditions. Immunofluorescence staining for Iba-1 revealed that MPTP treatment was associated with increased density and hypertrophic morphology of Iba-1-positive microglia in Cas9^Ctrl^ mice, whereas administration of plasma-derived EVs enriched with renal injury markers further enhanced these inflammatory alterations (Fig. 7b-f). In contrast, NEK7^KO+/–^ mice exhibited significantly reduced Iba-1 immunoreactivity and retained more resting-like microglial morphology under both MPTP and MPTP + EV conditions, suggesting attenuation of EV-associated microglial activation following partial loss of NEK7. Consistent with these findings, cytokine profiling demonstrated that MPTP and MPTP + EV treatments were associated with marked increases in pro-IL-1β and IL-1β levels in Cas9^Ctrl^ mice, whereas these inflammatory responses were significantly attenuated in NEK7^KO+/–^ mice (Fig. 7g-h). The reduction was more prominent for mature IL-1β than for pro-IL-1β, supporting the involvement of NEK7-associated inflammasome signaling in EV-related inflammatory responses.

**Fig. 8:**
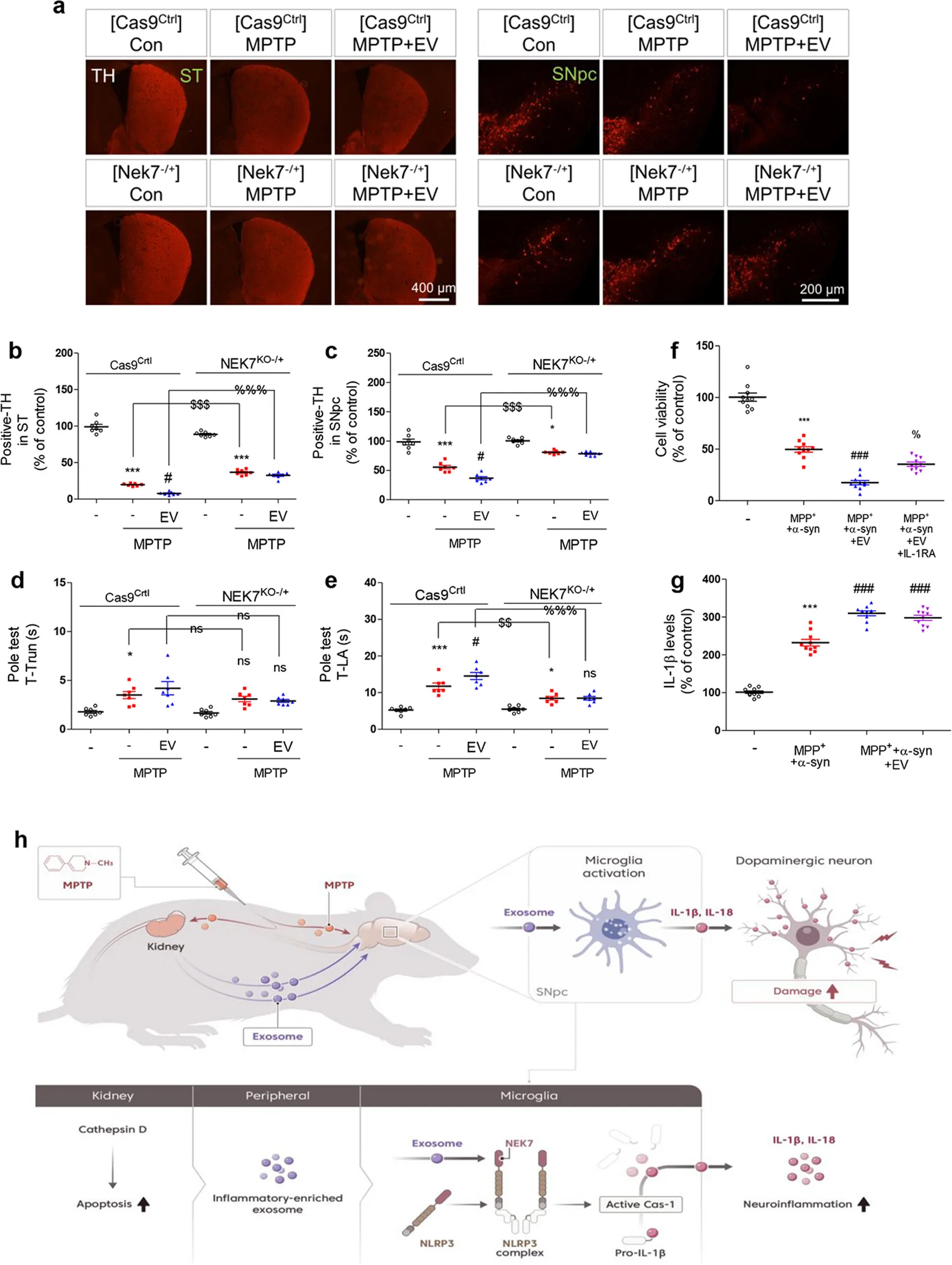
NEK7 deficiency attenuated EV-mediated dopaminergic neurodegeneration and behavioral impairment in the MPTP model. **a** Representative tyrosine hydroxylase (TH) immunofluorescence images in the striatum (ST) and substantia nigra pars compacta (SNpc) of Cas9^Ctrl^ and NEK7^KO/−+^ mice under Control, MPTP, and MPTP + EV treatment conditions. Scale bars: 400 μm (ST) and 200 μm (SNpc). **b-c** Quantitative analysis of TH-positive dopaminergic neuronal signals in the ST and SNpc, respectively. MPTP treatment markedly reduced TH-positive neuronal signals, whereas additional EV administration further exacerbated dopaminergic neuronal loss in Cas9^Ctrl^ mice. These effects were significantly attenuated in NEK7^KO/−+^ mice. **d-e** Pole test assessment of motor dysfunction, including turning latency (T-turn) and locomotor activity latency (T-LA). MPTP and EV co-treatment impaired motor performance, while NEK7 deficiency partially ameliorated these behavioral deficits. **f** Cell viability analysis in BV2 microglial cells treated with MPP^+^, α-synuclein, EVs, and IL-1 receptor antagonist (IL-1RA). Combined treatment with MPP^+^, α-synuclein, and EVs significantly reduced cell viability, whereas IL-1RA partially restored cell survival. **g** Measurement of IL-1β production in BV2 microglial cells following combined inflammatory stimulation. EV co-treatment further enhanced IL-1β expression in MPP^+^ and α-synuclein-treated conditions. **h** Proposed working model illustrating CTSD-associated renal stress, extracellular vesicle (EV)-mediated peripheral-to-brain inflammatory communication, NEK7-NLRP3 inflammasome activation, and downstream neuroinflammatory responses following systemic MPTP exposure. Data are presented as mean ± SEM. Statistical significance was determined using one-way ANOVA followed by Tukey’s post-hoc multiple comparison test. ^*^P < 0.05 and ***P < 0.001 versus the corresponding control group; ^#^*P* < 0.05 and ^###^*P* < 0.001 versus the indicated comparison group; ^%^*P* < 0.05 and ^%%%^*P* < 0.001 versus the Cas9^Ctrl^ MPTP + EV group; ^$$^*P* < 0.01 and ^$$$^*P* < 0.001 for the indicated comparisons

To further determine whether attenuation of inflammatory signaling was associated with preservation of dopaminergic neuronal integrity and behavioral outcomes, TH expression and motor behavioral analyses were subsequently performed. TH immunoreactivity in the substantia nigra pars compacta (SNpc) and striatum (ST) demonstrated that dopaminergic neuronal loss associated with MPTP and MPTP + EV exposure was substantially attenuated in NEK7^KO+/–^ mice compared with Cas9^Ctrl^ mice (Fig. 8a-c). Behavioral assessment using the pole test further demonstrated prolonged turning latency and locomotor latency following MPTP and EV exposure in Cas9^Ctrl^ mice, whereas these behavioral alterations were partially attenuated in NEK7^KO+/–^ mice (Fig. 8d-e). To additionally investigate whether EV exposure may cooperate with pathological α-syn-associated inflammatory signaling, BV2 microglial cells were treated with MPP^+^ (400 μM) and α-syn (10 μM), with or without plasma-derived EVs isolated from MPTP-treated mice. Cell viability assays demonstrated that MPP^+^ + α-syn treatment significantly reduced BV2 cell viability, which was further decreased by EV co-treatment (Fig. 8f). However, co-application of the IL-1 receptor antagonist (IL-1RA, 10 μg/mL) partially restored cell viability, suggesting involvement of IL-1-dependent inflammatory signaling. In parallel, combined treatment with MPP^+^ and α-syn significantly increased IL-1β expression, and co-treatment with MPTP-derived EVs further enhanced this response (Fig. 8g), suggesting that EV exposure may potentiate α-syn-associated inflammatory signaling. Together, these findings support the association of NEK7 signaling with EV-associated neuroinflammatory, dopaminergic, and motor-related pathological alterations following systemic MPTP exposure. In addition, EV exposure further amplified α-syn-associated inflammatory signaling and cytotoxic responses under the present experimental conditions. However, because the present study utilized a whole-body NEK7^KO+/–^ model, the relative contributions of glia-specific versus systemic immune effects could not be fully distinguished. To provide an integrated overview of the proposed mechanism based on our findings, a schematic working model summarizing the renal stress-associated peripheral-brain inflammatory axis is presented in Fig. 8h.

## Discussion

In this study, we introduce the concept of a renal injury-associated inflammatory pathway contributing to brain inflammation, in which MPTP-induced systemic toxicity extends beyond dopaminergic neurodegeneration to involve kidney-associated inflammatory signaling linked to the CNS. The active metabolite MPP⁺ accumulated in the kidneys, where increased CTSD enzymatic activity was associated with tubular injury and inflammatory alterations. Pharmacological inhibition of CTSD using pepstatin A markedly attenuated renal damage and inflammatory responses, supporting a potential association between CTSD-related lysosomal stress and altered extracellular vesicle (EV)-associated inflammatory signaling. These findings are consistent with a model in which renal stress-associated EV signaling may contribute to peripheral-central inflammatory communication, rather than establishing a single-organ-exclusive origin. Because renal tubular stress shares several features with acute kidney injury (AKI), similar lysosomal stress-associated inflammatory mechanisms may also operate in broader AKI-related inflammatory contexts.

Lysosomal dysfunction has been implicated in EV secretion and inflammatory cargo remodeling [26, 27]. Consistent with these observations, our findings suggest that CTSD-associated lysosomal stress may contribute to altered EV-associated inflammatory signaling under MPTP-induced renal injury conditions. We found that CTSD may participate in lysosomal stress and EV-mediated inflammatory signaling during AKI. Inhibition of lysosomal proteases by pepstatin A improved renal function, reduced apoptosis, and alleviated tubular injury in nephrotoxic and ischemia–reperfusion–induced AKI [28, 29]. Pepstatin A treatment also slowed interstitial fibrosis progression after ischemia–reperfusion [29, 30]. Therefore, CTSD, rather than serving as a primary pathogenic driver, may act as a potential mediator linking lysosomal stress to inflammatory EV release in AKI. Morphologically, EVs derived from injured tubular cells were more abundant and exhibited a reduced negative surface charge (zeta potential of–15 mV), consistent with an unstable, proinflammatory phenotype [31]. Moreover, TEM and NTA revealed that MPTP-treated or AKI-associated conditions were accompanied by increased EV size heterogeneity, broadening the particle distribution from 100 to 300 nm and increasing the relative abundance of larger vesicle populations. Although these larger particles may reflect stress-associated vesiculation or aggregation, their precise origin and biological significance remain unclear. Such physicochemical remodeling of EVs, including increased size and decreased surface charge, may influence EV-associated molecular composition and inflammatory activity, potentially contributing to enhanced microglial activation within a proposed renal-brain inflammatory framework [32–34]. Thus, pepstatin A-sensitive lysosomal protease activity involving CTSD may be associated with AKI-related inflammatory alterations through lysosome-associated inflammatory alterations and EV-related signaling, providing a possible mechanistic framework for future investigation of the lysosome-EV axis in renal injury.

MPTP exposure disrupted BBB integrity, potentially permitting circulating EV-associated signaling to influence the CNS [8, 25]. BBB breakdown was confirmed by Evans Blue extravasation, claudin-5/occludin disruption, and IgG leakage, consistent with multiple previous studies demonstrating that MPTP induces BBB dysfunction and vascular permeability alterations. Intravenous administration of MPTP-derived EVs, or direct striatal injection, markedly enhanced Iba-1-positive microglial activation and morphological hypertrophy, whereas co-treatment with the EV biogenesis inhibitor GW4869 significantly alleviated this response [35–37]. Furthermore, transient BBB modulation via cereport infusion produced generally comparable inflammatory changes, further supporting the possibility that circulating EVs enriched with renal injury markers may contribute to neuroinflammatory responses following systemic MPTP exposure. However, the precise relative contribution of EV-associated inflammatory signaling compared with the direct neurotoxic effects of MPTP could not be definitively quantitatively distinguished within the current experimental framework.

To elucidate the molecular mechanism underlying EV-induced inflammation, we focused on the NEK7-NLRP3 inflammasome axis. EVs carry damage-associated proteins and nucleic acids that can activate innate immune signaling [33]. This interpretation is consistent with previous reports demonstrating that extracellular vesicles can modulate inflammasome signaling in immune cells, including microglia and macrophages, without identification of a single responsible cargo molecule [38–40]. Previous studies have established NEK7 as a critical licensing factor for NLRP3 inflammasome activation [41–45]. Consistent with this framework, siRNA-mediated suppression of either NEK7 or NLRP3 attenuated EV-associated IL-1β responses in BV2 microglial cells, supporting the involvement of NEK7–NLRP3-associated signaling in EV-mediated inflammatory activation. NEK7-deficient mice were produced using CRISPR/Cas9 technology, and we confirmed embryonic and early postnatal lethality, consistent with previous reports, e.g., O’Regan and Fry; they showed that the complete loss of NEK7 disrupts mitotic progression, leading to metaphase arrest, chromosomal lagging, micronuclei formation, cytokinesis failure, and aneuploidy, ultimately resulting in developmental failure [46]. Similarly, in our CRISPR-generated NEK7^KO−/−^ line, most homozygous neonates showed marked growth retardation and died within 2–3 weeks after birth, rarely surviving beyond 1 month. We thus used heterozygous NEK7^KO+/–^ mice for in vivo experiments. Despite the essential role of NEK7 in cell division and organismal growth, partial reduction of NEK7 expression provided a physiologically viable model to examine its regulatory role in inflammasome activation. In these mice, MPTP- and EV-induced microglial activation and IL-1β expression were significantly attenuated, supporting the involvement of NEK7 in proinflammatory signaling associated with circulating EV exposure. Given the established role of NEK7 in inflammasome activation and neuroinflammatory signaling, the present findings support the potential involvement of NEK7 in EV-associated inflammatory responses [47–49]. However, because the present study utilized a whole-body NEK7^KO+/–^ model, the relative contributions of glia-specific versus systemic immune effects could not be fully distinguished. Moreover, dopaminergic neuronal loss in the substantia nigra and striatum was markedly reduced in NEK7^KO+/–^ mice, supporting the involvement of NEK7 in EV-associated neuroinflammatory and neurodegenerative responses following MPTP exposure. Consistently, NEK7 haploinsufficiency also partially attenuated MPTP- and EV-associated motor behavioral alterations in the pole test, supporting the potential functional relevance of EV-associated inflammatory signaling. However, direct analyses of inflammasome assembly were beyond the scope of the present study and warrant further investigation.

In vitro, EVs synergistically enhanced α-syn-induced IL-1β-mediated inflammatory responses. Co-treatment of BV2 cells with MPTP and α-syn markedly increased IL-1β expression, which was further enhanced by MPTP-derived EVs. IL-1RA addition partially rescued cell viability, suggesting that EV-induced cytotoxicity was mediated via IL-1-dependent pathways. These results suggest that peripheral EV-associated signaling may interact with α-syn pathology to exacerbate central inflammasome activation.

Our study has several limitations. First, the renal association of circulating EVs was inferred based on injury-marker enrichment rather than directly demonstrated through tissue-specific EV tracing or biodistribution analyses, and the precise inflammatory cargo contained within these EVs remains undefined. Furthermore, we did not determine whether the isolated EVs contained residual MPTP/MPP + or inflammatory mediators such as prostaglandins, cytokines, or other bioactive molecules that may contribute to the observed inflammatory responses. Therefore, future studies employing lineage-tracing, EV-tracking, proteomic, transcriptomic, metabolomic, and lipidomic approaches will be necessary to further clarify EV origin, cargo composition, and the molecular mediators responsible for the observed inflammatory effects. Second, although pepstatin A attenuated renal injury, EV alterations, and inflammatory responses, pepstatin A is not exclusively selective for CTSD and may inhibit additional lysosomal aspartic proteases. Accordingly, the present findings should be interpreted as supporting the involvement of pepstatin A-sensitive lysosomal protease activity rather than definitive CTSD-specific mechanisms, warranting future validation using CTSD-deficient or tissue-specific genetic models. Third, because the present study utilized a whole-body NEK7^KO+/–^ model, glia-specific and systemic immune contributions could not be fully distinguished. Future studies employing conditional or cell-specific genetic models will therefore be necessary to clarify the cell-type-specific role of NEK7 in neuroinflammatory processes. Fourth, the acute MPTP model, exploratory BBB analyses, and experimental approaches including stereotactic EV administration and BBB modulation may not fully recapitulate endogenous EV-associated communication or chronic PD pathology in vivo. Finally, although the present findings support a contribution of EV-associated inflammatory signaling to neuroinflammatory responses, the relative contribution of peripheral EV-mediated effects versus direct MPTP neurotoxicity could not be quantitatively distinguished within the current experimental framework. In addition, formal a priori power calculations were not performed in the present study.

In summary, our findings support a potential pathological framework involving CTSD-associated renal stress responses, EV signaling, NEK7-NLRP3 inflammasome activation, and IL-1β-associated neuroinflammatory changes. This study supports a renal injury-associated peripheral-brain inflammatory axis that provides a possible mechanistic framework for how systemic inflammation or renal dysfunction can exacerbate neurodegeneration in PD. NEK7 suppression more effectively attenuated IL-1β activation than NLRP3 silencing, suggesting that NEK7 may function as an important licensing factor in inflammasome assembly. Pharmacological modulation of the NEK7-NLRP3 interface, rather than direct NLRP3 inhibition, may provide a more selective means to restrain neuroinflammatory signaling. However, given NEK7's role in mitosis, future research should develop brain-targeted or interface-specific inhibitors to minimize systemic toxicity. As summarized in the proposed working model (Fig. 8h), targeting CTSD-associated lysosomal stress, EV release, or NEK7-NLRP3 inflammasome signaling may represent promising therapeutic strategies for mitigating neuroinflammation-associated neurodegeneration in PD.

## Materials and methods

### Cell culture, inflammatory stimulation, and siRNA transfection

BV2 microglia cells (#ABC-TC212S; AcceGen Biotechnology, Fairfield, USA) were maintained in Dulbecco’s Modified Eagle medium supplemented with 10% heat-inactivated fetal bovine serum and 1% penicillin/streptomycin at 37 °C under 95% air and 5% CO2. Cell line identity was confirmed by short tandem repeat (STR) profiling using a commercially available kit, and all cultures were tested negative for mycoplasma contamination using a commercial detection kit (Fig. S5). For inflammatory stimulation experiments, BV2 microglia cells were treated with MPP + (400 μM), α-synuclein (α-syn 10 μM) for 12 h unless otherwise indicated, and plasma-derived EVs isolated from MPTP-treated mice (15 μg/mL) for 24 h unless otherwise indicated. BV2 microglial cells were pretreated with IL-1 receptor antagonist (IL-1RA, 10 μg/mL) for 2 h before treatment with MPP +, α-synuclein, and plasma-derived EVs. For CTSD activity assays, HK-2 (#22,190; KCLB, Seoul, Korea) and TKPTS (#CRL-3361; ATCC, Manassas, VA, USA) renal tubular cells were treated with MPP + (400 μM) and harvested at the indicated time points (3, 6, 12, 24, and 48 h) for enzymatic activity analysis. BV2 microglia cells at 70–80% confluency were seeded in 100 mm dishes and transfected with siRNAs using Lipofectamine 2000. Cells were transfected with stealth siRNA targeting NLRP3, NEK7, CTSD or scrambled control siRNA using Lipofectamine 2000 (5 μL) mixed with 30 nM siRNA in 3 mL Opti-MEM (Gibco, Waltham, MA, USA). After incubation at 25 °C for 30 min, serum-free medium (500 μL) was added to each dish and cells were incubated for 24 h. Following transfection, BV2 microglia cells were subsequently treated with EVs for an additional 24 h.

### Molecular docking and SPR analysis

Molecular docking was performed to evaluate the binding interactions of MPP + with CTSD using AutoDock Vina and Schrödinger Glide software. Detailed docking parameters, software settings, and analytical procedures are described in detail in the Supplementary Methods section.

### Animals and drug administration

Male C57BL/6 mice (8-weeks old, weighing 23–24 g) were purchased from Doo Yeol Biotech (Seoul, Korea) and maintained under a 12 h light/dark cycle at 20–23 °C, with food and water provided ad libitum. Mice were randomly assigned to multiple experimental groups (*n* = 7 per group) to evaluate the effects of MPTP, pepstatin A, GW4869, cereport, and EV administration. MPTP (30 mg/kg/day, i.p.) was administered for 5 consecutive days to induce a subacute PD model. Pepstatin A (20 mg/kg/day, i.p.), GW4869 (3 mg/kg/day, i.p.), and EVs (100 µg, i.v.) were dissolved in saline and administered once per day for 5 days, with control animals received vehicle treatment. Cereport was infused via the internal carotid artery at a rate of 0.005 mL/min for 15 min (total volume: 0.75 mL). Additional groups received EVs alone, EVs combined with MPTP, or MPTP combined with GW4869 to evaluate interactions between EV-associated peripheral signaling and neuroinflammatory responses. For intracerebral delivery, mice were anesthetized and secured in a stereotaxic apparatus. EVs (10 μg/3 μL) were injected into the striatum over 5 min using a Hamilton microsyringe fitted with a 26-gauge needle. Stereotaxic coordinates were 0.5 mm posterior, 1.0 mm lateral to bregma, and 2.0 mm ventral to the skull surface. The needle remained in position for an additional 5 min after injection. The incision was closed with black silk, and the animals were allowed to recover under warming conditions. NEK7KO +/− mice were generated using a CRISPR/Cas9-based genome editing strategy targeting the NEK7 locus. Detailed sgRNA design, embryo manipulation procedures, genotyping strategy, and validation methods are described in the Supplementary Methods section (Fig. S6). For NEK7-associated in vivo experiments, mice were divided into six groups (*n* = 7 per group): (1) Cas9Ctrl-Con, (2) Cas9 Ctrl -MPTP, (3) Cas9 Ctrl -MPTP + EV, (4) NEK7KO +/− -Con, (5) NEK7KO +/− -MPTP, and (6) NEK7KO +/− -MPTP + EV. EVs (100 µg, i.v.) and MPTP (30 mg/kg, i.p.) were administered once per day for 5 consecutive days, while control mice received equal volumes of vehicle.

### EV isolation from mouse plasma, nanoparticle tracking analysis (NTA), and zeta potential and transmission electron microscopy (TEM)

EVs were isolated from platelet-depleted mouse plasma using size-exclusion chromatography followed by centrifugal concentration. EV size distribution, particle concentration, zeta potential, and ultrastructural morphology were subsequently analyzed using NTA, ZetaView-based electrophoretic mobility measurements, and TEM imaging. Detailed EV isolation procedures, acquisition parameters, instrument settings, and analytical conditions are described in detail in the Supplementary Methods section.

### Behavior, brain tissue preparation, immunohistochemistry, and ELISA

Behavior tests were conducted according to previously published methods [50, 51]. At 1 and 7 days after saline or MPTP treatment, mice were anesthetized and transcardially perfused with 0.05 M PBS followed by cold 4% PFA. Brains were extracted, post-fixed overnight, and cryoprotected in 30% sucrose. Coronal sections (30 μm) were cut using a freezing microtome and stored in cryoprotectant until immunohistochemistry (IHC). IHC was performed on sections containing the SNpc and striatum, sampled at standard bregma levels. Sections were incubated overnight at 4 °C with TH (1:2000) or Iba-1 (1:500) antibodies in buffer containing Triton X-100, BSA, and NGS, followed by Alexa-594-conjugated secondary antibodies for 1 h at room temperature. After mounting, sections were dehydrated, cleared, and coverslipped with fluorescence medium. TH-positive neurons in the SNpc and fiber density in the striatum were quantified using Stereo-investigator, and microglial activation was analyzed at the same magnification. Images were obtained with a BX53 microscope and quantified using Image J.

For ELISA, BV2 cells, supernatants, or SNpc tissue were homogenized and centrifuged; supernatants were stored at −80 °C. Cytokine levels were quantified using commercial ELISA kits following the manufacturer’s instructions.

### Statistical analysis

All statistical analyses were conducted using GraphPad Prism 7.0 software (GraphPad Software, San Diego, CA, USA). Data are expressed as mean ± standard error of the mean (SEM). Animals were randomly assigned to experimental groups whenever applicable, and quantitative analyses were performed under blinded conditions where feasible. Statistical analyses were performed using one-way ANOVA followed by Tukey’s post hoc test for multiple comparisons unless otherwise specified. Statistical significance is reported at *p* < 0.05.

## Supplementary Information

Supplementary Material 1.

Supplementary Material 2.

## Data Availability

The datasets generated and/or analyzed during the current study are available from the corresponding author upon reasonable request.
