# Supplementary material for: Renal stress-associated extracellular vesicle signaling linked to lysosomal cathepsin D and NEK7–NLRP3 neuroinflammation
Source: Mol Biomed. 2026 Oct 12;7:205. doi: 10.1186/s43556-026-00608-z (PMC13658691; doi:10.1186/s43556-026-00608-z)
Supplement: Supplementary file 1 [file 43556_2026_608_MOESM1_ESM.docx]

**[Supplementary methods, figure, table, and video]**

**Renal stress-associated extracellular vesicle signaling linked to lysosomal cathepsin D and NEK7–NLRP3 neuroinflammation**

**Materials and methods**

**Chemicals**

Paraformaldehyde (PFA), sodium chloride, sucrose, ethanol, DMSO, MPTP, MPP^+^, hydrogen peroxide, PBS, and sodium citrate buffer were obtained from Sigma-Aldrich (St. Louis, MO, USA). MPP^+^-d_3_ (iodide) and recombinant His-tagged cathepsin D (CTSD) were purchased from Chemscene (NJ, USA). Secondary antibodies, normal goat serum, and the VECTASTAIN Elite ABC kit were from Vector Laboratories (Burlingame, CA, USA). Anti-TH antibodies were obtained from Millipore (Bedford, MA, USA), and anti-Iba-1 antibodies from WAKO (Osaka, Japan). ELISA kits for IL-1β and pro-IL-1β were purchased from BioLegend (San Diego, CA, USA) and Biomatik (Ontario, Canada). DNA ladders, nuclease-free water, and nucleic acid stains were obtained from Invitrogen (Paisley, UK) and BD TransLabs (Franklin Lakes, NJ, USA). All other reagents were of analytical grade.

**Molecular docking and SPR analysis**

Molecular docking was performed to evaluate the binding interactions of MPP^+^ with CTSD using AutoDock Vina and Schrödinger Glide software. Briefly, the crystal structures of the CTSD (Protein Data Bank [PDB]: 1LYB) were retrieved from the PDB. For docking analysis, ligand and protein structures were prepared using CB-dock2; BIOVIA Discovery Studio 2024 was used for grid box generation and ligand optimization. Compounds were docked against CTSD using AutoDock Vina and Schrödinger Glide in standard precision mode, followed by extra precision rescoring. Binding affinities were ranked based on virtual screening scores, and interactions with key residues were analyzed. The highest-ranked binding pockets were characterized using the SiteMap module in Schrödinger to predict druggable regions. Molecular docking analysis results were further validated via surface plasmon resonance (SPR) analysis to confirm the predicted binding interactions. Then, SPR analysis was performed to investigate the binding kinetics and affinity between CTSD and MPP⁺ using a Biacore T200 system (Cytiva, Uppsala, Sweden) at the Chiral Material Core Facility Center, Sungkyunkwan University. The assay was conducted at 25 °C using a CM5 sensor chip (Cytiva, Cat. No. 29149603). The ligand, CTSD (DL form, 44.5 kDa), was prepared at a stock concentration of 10 mg/mL in 2.5 mM MES buffer containing 75 mM NaCl (pH 6.5) and 50% glycerol as protectant, and diluted to 7 µg/mL in 10 mM sodium acetate buffer (pH 5.5) before immobilization. The chip surface was activated using EDC/NHS chemistry, and the ligand was immobilized to approximately 3934 RU. Unreacted active groups were blocked with 1 M ethanolamine (pH 8.5). PBS with 3 mM EDTA (pH 7.4) was used throughout the assay. For the binding experiments, MPP⁺ (297.13 Da) was prepared at 1 mg/mL in PBS (pH 7.4) and further diluted as follows: (1) PBS with 3 mM EDTA, (2) PBS with 3 mM EDTA and 0.5 mM tris(2-carboxyethyl)phosphine, or (3) PBS with 3 mM EDTA adjusted to pH 6.4. MPP⁺ was injected over the immobilized CTSD surface at a flow rate of 30 µL/min for 180 s, with a dissociation time of 300 s; association and dissociation phases were monitored under continuous flow conditions. Between binding cycles, the chip surface was regenerated using 750 mM NaCl to remove residual analyte and restore baseline activity. Real-time sensorgrams were obtained; nonspecific binding was corrected by subtracting the response from a reference channel. Binding parameters, including the association rate constant (kₐ), dissociation rate constant (k_d), and equilibrium dissociation constant (K_D), were calculated using a 1:1 Langmuir binding model implemented in Biacore Evaluation Software (v3.2, Cytiva). All experiments were conducted in triplicate; representative data are presented.

**Generation of NEK7 heterozygous knockout (NEK7^KO(-/+)^) mice using CRISPR/Cas9**

To generate NEK7^KO(-/+)^ mice, sgRNA targeting exon 5 of the NEK7 gene and Cas9 protein tagged with a nuclear localization signal were purchased from Macrogen, Inc. sgRNA activity was validated using *in vitro* cleavage reactions. Amplified NEK7 DNA was incubated with 20 nM of Cas9 protein and 40 nM of sgRNA in 1× NEB 3.1 buffer at 37 °C for 90 min. Reactions were terminated using a 6× stop solution containing 30% glycerol, 1.2% SDS, and 100 mM EDTA. Cleavage activity was confirmed via electrophoresis of the reaction mixtures. To generate NEK7^KO(-/+)^ mice, CRISPR/Cas9-mediated gene editing was performed targeting exons 6–7 of the NEK7 gene (NM_021605.5) (Fig. G). Two guide RNAs were designed within intron 5 and intron 7, respectively: NEK7_int5_gR1 (5′ -GGGCAAGCTTACTTCAGCGAGG-3′) and NEK7_int7_gR1 (5′-ACACACGTAACGGACTGAAGCGG-3′). PCR amplification across the target region generated a 778 bp fragment using primers F1 (5′-GGTGTTTCAGGTGACCCATGC-3′) and F2 (5′-GTAAGAGGGAACTGTGAGCACCCAAACGGTTCTCTCAGGGAT-3′) (Fig. 4H). Fertilized embryos were collected from female C57BL/6N mice that had been superovulated with PMSG and HCG and mated with C57BL/6N stud males. Females displaying vaginal plugs were killed to harvest fertilized embryos. The sgRNA and Cas9 protein mixture was microinjected into one-cell stage embryos, which were subsequently incubated at 37 °C for 1–2 h before transplantation into oviducts of pseudopregnant ICR recipient mice. The resulting heterozygous NEK7^KO(-/+)^ mice were maintained on a C57BL/6 background without further backcrossing because of reduced viability during homozygous breeding (Fig. S1).

**Quantification of MPP^+^ in mouse kidneys**

MPP**^+^** levels in mouse kidneys were measured by LC–MS/MS (Life Science Laboratory eMass, Seoul, Korea) following the ICH M10 bioanalytical guideline. Kidney tissues were homogenized in five volumes of 50% methanol/0.1% formic acid; 50 μL homogenate was extracted with solvent containing MPP**^+^**-d_3_ (internal standard, 10 ng/mL). After vortexing and centrifugation (13,000 rpm, 10 min, 4 °C), supernatants were transferred to vials, and 2 μL was injected for analysis. Chromatography was performed on a Kinetex C18 column using 0.1% formic acid in water (A) and acetonitrile (B) at 0.3 mL/min. The API 4000 mass spectrometer operated in positive ESI mode with MRM transitions 170.3 → 127.0 (MPP**^+^**) and 173.3 → 154.1 (MPP**^+^**-d_3_). Calibration curves (0.1-1000 ng/mL) were linear (r ≥ 0.999; LLOQ 0.1 ng/mL). Quantification was performed using peak area ratios with a 1/x² weighted regression model. Samples, MPP**^+^** was undetectable in controls, whereas experimental tissues contained 407-3090 ng/mL, confirming substantial renal accumulation after systemic administration.

**EV isolation from mouse plasma, Nanoparticle tracking analysis (NTA), and Zeta potential and transmission electron microscopy (TEM)**

Whole blood was placed in EDTA tubes and processed within 30 min at 4 °C. Platelet-depleted plasma was prepared via sequential centrifugations at 2,000 × *g* for 15 min and 10,000–13,000 × *g* for 20 min, followed by 0.22 µm filtration. EVs were isolated using size-exclusion chromatography (qEVoriginal, 70 nm cutoff); vesicle-enriched fractions were pooled and concentrated using 100-kDa MWCO centrifugal filters. Preparations were stored on ice and analyzed on the same day. Also, EV size and concentration were measured on a ZetaView instrument (Particle Metrix) equipped with a 488-nm laser. Samples were diluted in PBS to approximately 100-200 particles/frame (typical dilution factor approximately 1:3000) and measured at 11 positions, 1 cycle per sample (video: 30 fps; resolution: high). Acquisition settings were: sensitivity 80, shutter 100; temperature 22.9 °C (measured), pH 7.0 (input), conductivity 15,000 µS/cm. Analysis parameters were: Max area 1000, Min area 10, Min brightness 25, bin width 5 nm/class, TL 15. Median diameter (X50), X10/X90, mean size, FWHM, span, and particle concentration (particles/mL) were exported with QC metrics (average counted particles per frame and number of traced particles) according to the reporting checklist. Software: ZetaView v8.06.01 SP1.Then, electrophoretic mobility measurements were performed on the same ZetaView platform in PBS (pH 7.0) at 22–23 °C. Zeta potential (mV) was computed by the instrument software using the Smoluchowski model; three technical videos were acquired at 11 positions and averaged per sample. EVs (3–5 µL) were adsorbed to glow-discharged carbon-coated copper grids for 1–2 min, washed with distilled water, and negatively stained with 2% (w/v) uranyl acetate for 1 min. Grids were air-dried and imaged on an 80–200 kV transmission electron microscope (TEM).

**Fig. S1.** In silico target prioritization and interaction-network analysis of MPP^+^-associated proteins. **a** The complete list of predicted targets is shown in tabular form, with prioritized candidates highlighted in blue. Cathepsin D (CTSD) was selected as a candidate target based on its high prediction probability, high model accuracy, available PDB structure, and network connectivity. **b** The protein-protein interaction network of selected targets was generated to visualize functional relationships among MPP⁺-associated candidates; CTSD is indicated by the red circle. Probability and model accuracy values for representative network-associated targets are annotated.

**Fig. S2.** Pepstatin A restores proximal tubular brush border integrity disrupted by MPTP. **a** Representative multicolor immunofluorescence images of renal proximal tubules from control, MPTP-, and MPTP + pepstatin A-treated mice. Higher-magnification views of the boxed regions are shown below. MPTP treatment induces marked disruption and disorganization of the apical brush border (merge signal), whereas pepstatin A treatment partially restores tubular structural integrity. Dashed lines indicate the regions used for fluorescence intensity profile analysis. Nuclei were counterstained with DAPI (blue). **b** Line-scan fluorescence intensity profiles corresponding to the dashed regions shown in a. MPTP-treated kidneys exhibit altered spatial distribution and reduced organization of fluorescence signals compared with controls, while pepstatin A treatment partially normalizes the signal pattern. Fluorescence intensity is presented as arbitrary units (a.u.) across the indicated distance (pixels).

**Fig. S3.** Identification and analysis of MPP⁺-associated gene signatures using Super-PRED. Gene targets potentially associated with MPP^+^ were identified using the Super-PRED (Supervised Chemical Classification and Target Prediction) tool. The top-ranked predicted genes were subjected to functional annotation and pathway enrichment analysis to explore biological processes linked to MPP⁺-induced toxicity.

**Fig. S4.** Proposed mechanism linking kidney-associated EVs to NEK7–NLRP3 inflammasome-associated neuroinflammation in the MPTP model. Systemic MPTP exposure induces renal CTSD-associated stress responses and inflammatory extracellular vesicle (EV) release. Under conditions of BBB disruption, circulating EVs promote microglial activation and NEK7–NLRP3 inflammasome signaling, leading to increased IL-1β/IL-18-associated neuroinflammation and dopaminergic neuronal damage.

**Fig. S5.** Mycoplasma contamination screening of the cell lines used in this study. Mycoplasma testing was performed using the MycoProbe Mycoplasma Detection Kit (R&D Systems, Catalog #CUL001B) according to the manufacturer’s instructions. Optical density (O.D.) values below 0.05 were considered negative for mycoplasma contamination. BV2, TKPTS, and HK-2 cells used in the present study were all confirmed to be negative for mycoplasma contamination.

**Fig. S6.** Genotyping strategy and PCR validation for heterozygous NEK7 knockout (NEK7^KO(-/+)^) mice. Schematic representation of the CRISPR/Cas9-mediated NEK7 targeting strategy, showing the guide RNA (sgRNA) sites and primer positions (F1, R1, R2) used for genotyping. Genomic PCR amplification was performed using specific primers (Forward: NEK7_F1, Reverse: NEK7_R1/R2) to detect wild-type (778 bp) and mutant alleles. PCR reactions were performed using Axen™ High-Q DNA Polymerase under optimized cycling conditions (98 °C 30 s; 35 cycles of 98 °C 10 s, 60 °C 10 s, 72 °C 1 min; final extension 72 °C 5 min). Representative agarose gel electrophoresis results (2%) show distinct bands corresponding to wild-type (300 bp) and heterozygous mutant alleles across multiple offspring. M, molecular weight marker; PC, positive control; NC, negative control.

**Table S1.** Predicted molecular targets of MPP^+^ identified by in silico target profiling.

| **Target Name** | **PDB Visualization** | **Probability** | **Model accuracy** |
| --- | --- | --- | --- |
| **Tyrosyl-DNA phosphodiesterase 1** | 6N0D | 93% | 71% |
| **Cathepsin D** | 4OD9 | 90% | 99% |
| **Transcription intermediary factor 1-alpha** | 4YBM | 88% | 96% |
| **Kruppel-like factor 5** | Not Available | 88% | 86% |
| **Muscarinic acetylcholine receptor M5** | 6OL9 | 86% | 95% |
| **Cyclooxygenase-1** | 6Y3C | 86% | 90% |
| **Muscarinic acetylcholine receptor M1** | 6OIJ | 84% | 94% |
| **Endoplasmic reticulum-associated amyloid beta-peptide-binding protein** | 2O23 | 82% | 70% |
| **Nuclear receptor ROR-beta** | Not Available | 82% | 95.5% |
| **DNA-(apurinic or apyrimidinic site) lyase** | 6BOW | 81.3% | 91% |
| **Monoamine oxidase B** | 1S3E | 81% | 93% |
| **Nuclear factor NF-kappa-B p105 subunit** | 1SVC | 81% | 96% |
| **G-protein coupled receptor 55** | Not Available | 79% | 78% |
| **HERG** | 5VA1 | 79% | 90% |
| **Bloom syndrome protein** | 4O3M | 77% | 70% |
| **Muscarinic acetylcholine receptor M4** | 5DSG | 76% | 94% |
| **Neprilysin** | 6SUK | 75% | 93% |
| **Geranylgeranyl pyrophosphate synthetase** | 6R4V | 75% | 92% |
| **Nuclear factor erythroid 2-related factor 2** | 2FLU | 73% | 96% |
| **Glutathione S-transferase Pi** | 5J41 | 73% | 94% |
| **Monoamine oxidase A** | 2Z5Y | 73% | 91% |
| **ADAM10** | 6BE6 | 73% | 97.5% |
| **Aminopeptidase N** | 4FYT | 73% | 93% |
| **Toll-like receptor 8** | 3WN4 | 72% | 96% |
| **GABA-A receptor; alpha-1/beta-2/gamma-2** | 6X3T | 70.7% | 93% |
| **Beta-glucosidase** | Not Available | 69.2% | 99% |
| **TRAF2- and NCK-interacting kinase** | 2X7F | 69% | 70% |
| **Free fatty acid receptor 1** | 5TZR | 68% | 93% |
| **Pregnane X receptor** | 6TFI | 68% | 95% |
| **Choline kinase alpha** | 4DA5 | 67% | 78% |
| **Endoplasmic reticulum aminopeptidase 1** | 6Q4R | 66% | 100% |
| **Dual specificity phosphatase Cdc25C** | 3OP3 | 66% | 97% |
| **Histamine H3 receptor** | Not Available | 64% | 91% |
| **Lipoxin A4 receptor** | 6OMM | 64% | 100% |
| **Voltage-gated N-type calcium channel alpha-1B subunit** | Not Available | 63% | 97% |
| **Potassium channel subfamily K member 9** | 6GHP | 63% | 80% |
| **Lysosomal Pro-X carboxypeptidase** | 3N2Z | 62% | 100% |
| **Formyl peptide receptor 1** | Not Available | 62% | 94% |
| **Dual specificity protein phosphatase 3** | 3F81 | 62% | 94% |
| **Glycine transporter 2** | Not Available | 60% | 99% |
| **Voltage-gated potassium channel subunit Kv1.5** | Not Available | 60% | 94% |
| **Histone acetyltransferase KAT6A** | 2OZU | 60% | 97.5% |
| **Proteasome component C5** | 6KWY | 60% | 90% |
| **Casein kinase II alpha/beta** | 6TLS | 59% | 99% |
| **TGF-beta receptor type II** | 5QIN | 59% | 88% |
| **Metabotropic glutamate receptor 5** | 6N4X | 59% | 96% |
| **Stimulator of interferon genes protein** | 6NT5 | 58% | 95% |
| **Angiotensin-converting enzyme** | 5AMB | 58% | 93% |
| **Phosphodiesterase 11A** | Not Available | 58% | 85% |
| **Trypsin I** | 2RA3 | 58% | 90% |
| **Calpain 1** | 1ZCM | 57% | 93% |
| **NADPH oxidase 1** | Not Available | 57% | 95% |
| **Inhibitor of nuclear factor kappa B kinase alpha subunit** | 5EBZ | 57% | 96% |
| **Tyrosine-protein kinase ITK/TSK** | 4HCU | 57% | 95% |
| **Integrin alpha-4/beta-1** | 3V4V | 56.4% | 93% |
| **Caspase-6** | 2WDP | 56% | 98% |
| **Fatty acid binding protein adipocyte** | 3P6D | 56% | 96% |
| **Peptidyl-prolyl cis-trans isomerase NIMA-interacting 1** | 1PIN | 56% | 92% |
| **Glyoxalase I** | 3W0T | 56% | 92% |
| **Plasminogen activator inhibitor-1** | 3CVM | 55% | 83% |
| **G protein-coupled receptor kinase 5** | 4TND | 55.2% | 88% |
| **Farnesyl diphosphate synthase** | 5CG5 | 55% | 92% |
| **Tyrosine-protein kinase FYN** | 2DQ7 | 54% | 81% |
| **Transmembrane protease serine 6** | Not Available | 54% | 98% |
| **Galectin-3** | 6FOF | 54% | 96.9% |
| **Proteasome subunit beta type-9** | 6E5B | 54.1% | 97.5% |
| **G-protein coupled bile acid receptor 1** | 7CFM | 54% | 94% |
| **Cysteine protease ATG4B** | 2CY7 | 54% | 87.5% |
| **Niemann-Pick C1 protein** | 6W5S | 53% | 81% |
| **Activin receptor type-1B** | Not Available | 53% | 70% |
| **Proteasome Macropain subunit** | 5LE5 | 53% | 90% |
| **Neurokinin 2 receptor** | Not Available | 53% | 99% |
| **Tissue factor pathway inhibitor** | 5NMV | 52% | 97% |
| **Dopamine D1 receptor** | 7JVP | 52% | 91% |
| **Histone deacetylase 4** | 2VQM | 52% | 93% |
| **Signal transducer and activator of transcription 1-alpha/beta** | 1YVL | 52% | 73% |
| **Muscarinic acetylcholine receptor M3** | Not Available | 51% | 98% |
| **Glutaminase kidney isoform, mitochondrial** | 3UO9 | 50% | 100% |
| **CaM-kinase kinase beta** | 6CMJ | 50% | 89% |

**Supplementary Video file**

**Supplementary Video.** **a** Nanoparticle tracking analysis (NTA) video showing the size distribution and motion of extracellular vesicles (EVs) isolated from plasma of MPTP-treated mice. Representative NTA recording demonstrates the concentration and Brownian motion of particles within the EV suspension, confirming a mean diameter of approximately 100–150 nm. The dynamic video corresponds to the quantitative NTA data presented in Fig. 5a. **b** Characterization of extracellular vesicles (EVs) isolated from control and MPTP-treated groups and detection of renal tubular-associated proteins in EV fractions. Representative immunoblot images showing the expression of EV markers (CD63, CD81, TSG101, and Alix), the negative marker GM130, and renal tubular-associated proteins (KIM1, AQP1, and LRP2) in cell lysates and isolated EV fractions from control (Cont) and MPTP-treated groups. EV fractions showed enrichment of canonical EV markers, whereas GM130 was predominantly detected in cell lysates, indicating minimal cellular contamination in isolated EV preparations. Renal tubular-associated proteins, including AQP1 and LRP2, were detected in EV fractions, and KIM1 expression was increased in EVs derived from the MPTP-treated group. Red boxes indicate cropped regions corresponding to the bands presented in the assembled blot images. Molecular weights of each target protein are indicated on the right.
